# Draft Genome Sequence of *Lactobacillus delbrueckii* subsp. *bulgaricus* CRL871, a Folate-Producing Strain Isolated from a Northwestern Argentinian Yogurt

**DOI:** 10.1128/genomeA.00693-15

**Published:** 2015-06-25

**Authors:** Jonathan Emiliano Laiño, Elvira María Hebert, Graciela Savoy de Giori, Jean Guy LeBlanc

**Affiliations:** a Centro de Referencia para Lactobacilos (CERELA-CONICET), San Miguel de Tucumán, Tucumán, Argentina; b Cátedra de Microbiología Superior, Facultad de Bioquímica, Química y Farmacia, Universidad Nacional de Tucumán, San Miguel de Tucumán, Tucumán, Argentina

## Abstract

*Lactobacillus delbrueckii* subsp. *bulgaricus* CRL871 is the first strain of *L. delbrueckii* subsp. *bulgaricus* reported as a folate-producing strain. We report the draft genome sequence of *L. delbrueckii* subsp. *bulgaricus* CRL871 (2,063,981 bp, G+C content of 49.1%). This strain is of great biotechnological importance to the dairy industry because it constitutes an alternative to folic acid fortification.

## GENOME ANNOUNCEMENT

*Lactobacillus delbrueckii* subsp. *bulgaricus* is a lactic acid bacterium (LAB) that is widely used as a starter culture in the dairy industry for the manufacture of fermented milk products, such as yogurt, when combined with *Streptococcus thermophilus*. Historically, *L. delbrueckii* subsp. *bulgaricus* was described as a folate consumer that normally took up folates produced by *S. thermophilus*. *L. delbrueckii* subsp. *bulgaricus* CRL871, isolated from an artisanal yogurt in the northwestern region of Argentina, showed the ability to produce folate when grown in folate-free culture medium (1) and also in milk (2). The ability to produce folate confers to this strain an important biotechnological ability, allowing the development of novel fermented milk products naturally bioenriched in folates, representing a viable, safe, and affordable biotechnological alternative to folic acid fortification. Here, we report the draft genome sequence of the first folate-producing *L. delbrueckii* subsp. *bulgaricus* strain described. The genome sequence of this strain allowed the identification of genes responsible for folate-producing enzymes and its future biotechnological application in food industry.

The genome of *L. delbrueckii* subsp. *bulgaricus* CRL871 was sequenced using a whole-genome shotgun (WGS) strategy (40-fold genome coverage, *N*_50_ contig length of 59,563 bp) with an Ion Torrent personal genome machine (Life Technologies). Quality filtered reads were assembled using the Ngen (DNASTAR) assembler, giving 63 contigs that are more than 2,000 bp in length. Genome annotation was done using the NCBI Prokaryotic Genome Annotation Pipeline (http://www.ncbi.nlm.nih.gov/genome/annotation_prok/) and from the Rapid Anotations using Subsystems Technology (RAST) server (3). tRNAs and rRNAs were identified by tRNAscan-SE and RNAmmer, respectively (4, 5).

The genome size consists of 2,063,981 bp with a mean G+C content of 49.1%. A total of 2,313 coding sequences (CDS), 1,932 genes with predicted function, 122 structural tRNAs, and 39 rRNA were predicted. Additionally, there are 264 RAST subsystems represented in the chromosome, which represent only 47% of the assigned sequences.

As was previously mentioned, *L. delbrueckii* subsp. *bulgaricus* CRL871 showed the ability to synthetize folates when grow in a folate-free culture medium (1) and in milk (2). Genomes from different strains of *L. bulgaricus* showed sequences coding enzymes responsible for folates synthesis. However, none of these strains have been described as folate producers. *In silico* genomic analysis of *L. delbrueckii* subsp. *bulgaricus* CRL871 revealed the presence of *folA*, *folC*, *folP*, and *phoA* genes, which are responsible for *de novo* synthesis of folates. The presence of these genes together with the ability to synthetize folate gives this strain a very important biotechnological potential in the dairy industry for the development of yogurt naturally bio-enriched in folate, as was recently reported (2, 6).

### **Nucleotide sequence accession number**s.

The *L. delbrueckii* subsp. *bulgaricus* CRL871 whole-genome shotgun project has been deposited at DDBJ/EMBL/GenBank under the accession no. JXRV00000000. The version described in this paper is version JXRV01000000.
